# Isolation and Characterization of Two Novel Plasmids from Pathogenic *Leptospira interrogans* Serogroup Canicola Serovar Canicola Strain Gui44

**DOI:** 10.1371/journal.pntd.0003103

**Published:** 2014-08-21

**Authors:** Wei-Nan Zhu, Li-Li Huang, Ling-Bing Zeng, Xu-Ran Zhuang, Chun-Yan Chen, Yan-Zhuo Wang, Jin-Hong Qin, Yong-Zhang Zhu, Xiao-Kui Guo

**Affiliations:** 1 Department of Immunology and Microbiology, Institutes of Medical Sciences, Shanghai Jiao Tong University School of Medicine, Shanghai, China; 2 Soochow University Affiliated Children's Hospital, Soochow, China; 3 The First Affiliated Hospital of Nanchang University, Nanchang, China; University of Tennessee, United States of America

## Abstract

**Background:**

Previous genomic analysis of pathogenic *Leptospira* has identified two circular chromosomes but no plasmid. This study aims to investigate potential extrachromosomal elements of *L.interrogans* serovar Canicola strain Gui44.

**Methodology:**

Two novel plasmids, pGui1 and pGui2, were isolated from the pathogenic strain Gui44, using a modified alkaline lysis method. Southern blotting was performed to determine the presence and size of them. Then, 454 and Hiseq sequencing were applied to obtain and analyze the complete sequences of the two plasmids. Furthermore, real-time quantitative PCR and next-generation sequencing were used to compare relative copy numbers of the two plasmids with that of the chromosomes. Finally, after serial passages *in vitro* for more than 2 years, the strain Gui44 was subsequently re-sequenced to estimate stability of the two plasmids.

**Principal Findings:**

The larger plasmid, pGui1, 74,981 base pairs (bp) in length with GC content of 34.63%, possesses 62 open reading frames (ORFs). The smaller plasmid, pGui2, is 66,851 bp in length with GC content of 33.33%, and contains 63 ORFs. The replication initiation proteins encoded by pGui1 and pGui2 demonstrate significant sequence similarity with LA1839 (86% and 88%), a well-known replication protein in another pathogenic *L.interrogans* serovar Lai strain Lai, suggesting the ability for autonomous plasmid replication. Quantitative PCR and next-generation sequencing confirms a single copy of both plasmids and their stable presence in the strain Gui44 with *in vitro* serial passages after more than 2 years. Interestingly, the two plasmids both contain a significant number of novel genes (35 in pGui1 and 52 in pGui2).

**Conclusions:**

This report confirms the presence of two separate circular plasmids in serovar Canicola strain Gui44 and provides a new understanding of genomic organization, adaptation, evolution and pathogenesis of *Leptospira*, which will aid in the development of *in vivo* genetic manipulation systems in pathogenic *Leptospira* species.

## Author Summary

*Leptospira* species are the causative agent of leptospirosis, one of the most common animal to human transmitted diseases. Previous genomic analysis of *L.interrogans* serovar Lai and Copenhageni has identified the presence of large (4.33 mega base) and small (350 kilo base) circular chromosomes without evidence of any plasmids. Detailed understanding of *Leptospira* and its pathogenicity was delayed by the lack of available genetic tools. In this study we confirm the existence of two novel plasmids in *L.interrogans* serovar Canicola strain Gui44, an epidemic strain in China. Some novel genes identified in the two plasmids may play important roles in the characterization of the strain. The two plasmids will provide useful information in understanding the diversity of *Leptospira* genome and markedly improve our understanding of the evolution and pathogenesis of *L.interrogans*. In particular, it will contribute to the development of genetic manipulation systems in pathogenic *Leptospira* species.

## Introduction

Leptospirosis is caused by spirochetes belonging to the genus *Leptospira*, which is comprised of both saprophytic and pathogenic species [1]. The spectrum of clinical symptoms of human leptospirosis ranges from asymptomatic to severe manifestation causing multi-organs dysfunction and even death [2]. There are eight known pathogenic species of *Leptospira*, and over 200 pathogenic serovars [3], [4]. Humans and animals become infected either through direct or indirect exposure to *Leptospira* spp. shed from the host organism via urine [5]. More than 500,000 cases of severe leptospirosis are reported each year, with mortality rates up to 23% in some outbreaks [6]. In addition, Leptospirosis remains recognized as a major veterinary disease worldwide as it infects a broad range of domestic animals, incurring large economic losses [5], [7].

Until recently, the lack of available genetic tools delayed the full characterization and understanding of pathogenic *Leptospira* and its pathogenicity [6]. Plasmid vectors have been successfully generated using the 54 kilo base (kb) genomic island LaiGI from the large chromosome of *L.interrogans* strain Lai, which has the ability to excise itself from the chromosome [8]. The plasmid vector can autonomously replicate in both *L.biflexa* and *E. coli*. In addition, DNA has been introduced into *Leptospira* by electroporation [8]. However, none of this work has led to the construction of a replicative plasmid vector for pathogenic *Leptospira*, and absence of naturally occurring plasmids in pathogenic *Leptospira* further impedes investigation of its biology and virulence mechanisms by classic genetic studies.

The recent whole genome sequencing of seven pathogenic *Leptospira* strains including *L.interrogans* serovar Lai strain Lai and its virulence-attenuated strain IPAV, serovar Copenhageni strains Fiocruz L1-130 and two virulent strains of *L.borgpetersenii* serovar Hardjo (strain L550 and JB197) [9]–[12], as well as two saprophytic, *L.biflexa* serovar Patoc strains Paris and Ames [13], has provided a better understanding of evolution, metabolism and pathogenicity of the organism. Based on these genomic data, *L.biflexa* is the first reported species in which a 74 kb extrachromosomal plasmid named p74 was identified [13]. Previous evidence had showed that all of the pathogenic *Leptospira* species sequenced only contained two chromosomes as well as one special 54 kb genomic island LaiGI in the large chromosome [14].

*L.interrogans* is the most frequently reported agent of leptospirosis and many serovars have adapted to a biased mammalian reservoir host, such as serovar Lai and Copenhageni (rodents), serovar Canicola (dogs) and serovar Hardjo (cattle) [15], [16]. Interestingly, focusing on one pathogenic serovar Lai strain 56601, our recent study re-analyzed the previously reported mobile, phage-related genomic island LaiGI in its large chromosome and first confirmed the presence of one separate circular extrachromosomal plasmid named Laicp in strain 56601 by a combination of various experimental methods [17]. Based on the finding, we continued to perform isolation and characterization of potential circular extrachromosomal elements in another pathogenic *L.interrogans* serovar Canicola strain Gui44, which was originally isolated from a patient with leptospirosis and was also one of the main epidemic strains in China (Institute for Infectious Disease Control and Prevention, Beijing, China). Surprisingly, in the present study, strain Gui44 was proved to possess two circular plasmids which were distinct from the simple one plasmid Laicp found in strain 56601. This report first described the two novel plasmids, pGui1 and pGui2, isolated from the pathogenic strain Gui44. Sequence analysis determined that the two plasmids contained novel genes, which may provide strain Gui44 with unique properties involved in adaptation and pathogenesis and give insight into the genome dynamics of this organism. To best of our knowledge, so far, except the pathogenic strain 56601 and Gui44, there was no other report of circular plasmid isolated from pathogenic *Leptospira*. Finally, these results may significantly contribute to our comprehensive understanding of the complicated diversity of pathogenic *Leptospira* genome and enable construction of a new shuttle vector and molecular tool to facilitate the investigation of its virulence mechanisms.

## Methods

### Bacterial strains and growth conditions

*L.interrogans* serogroup Canicola serovar Canicola strain Gui44 was obtained from the Institute for Infectious Disease Control and Prevention, Beijing, China. The strain is grown at 28°C in liquid Ellinghausen-McCullough-Johnson-Harris (EMJH) medium under aerobic conditions and collected at mid-log phase [18].

### Isolation of plasmids

Plasmids of strain Gui44 were isolated using a previously published alkaline lysis method with some modifications [19], [20]. During mid-log growth phase, 500 mL of cells were collected by centrifugation (8000 rpm for 20 min at 4°C) and washed twice in 100 mL Tris-EDTA (TE) buffer (10 mM Tris–Cl, 1 mM EDTA, pH 8.0). Cells were then resuspended in 10 mL of solution I (2 mg/ml lysozyme, 10% sucrose, 20 mM Tris–Cl, 1 mM EDTA, RNase 100 µg/ml, pH 8.0) and incubated at 37°C for 2 h. Cells in solution I were then mixed with 20 ml of solution II (0.2 M NaOH, 1% SLS) and incubated for 3 min on ice. Cells were treated with 15 mL of solution III (sodium acetate, pH 4.8) and incubated for 5 min on ice followed by centrifugation at 12,000 rpm for 30 min. DNA was precipitated with 38% isopropanol, washed twice with 70% ethanol, and dissolved in sterile water. Total genomic DNA of strain Gui44 was extracted by the phenol-chloroform method as previously described [21].

### Sequencing and sequence analysis of total genomic and plasmid DNA of strain Gui44

In March 2009, the genome of *L.interrogans* serovar Canicola strain Gui44 was first sequenced using 454 GS 20 sequencing system (454 Life Science Corporation, Branford, CT, USA) at the Chinese National Human Genome Center (CHGC, Shanghai, China). After physical gap closing, the complete genome of the strain contained two circular chromosomes (unpublished data) and two novel extrachomosomal elements. Based on this finding, plasmid DNA and total genomic DNA of strain Gui44, respectively, were obtained to further perform whole genome re-sequencing with Hiseq 2000 sequencing system (Illumina, San Diego, CA, USA) in September 2011 at CHGC. According to the complete sequences of the two plasmids by 454 sequencing, the nucleotide sequences of two plasmids were assembled into 16 contigs larger than 1 kb. The physical gaps were closed by sequencing gap-spanning PCR products or clones using ABI 3730×l DNA Analyzer. PCRs were performed under the following conditions: 5 min at 95°C, followed by 35 cycles of 30 sec at 95°C, 30 sec at 56°C, and 30 sec at 72°C. Sequence assembly was accomplished by using the Phred/Phrap/Consed software package. Sequencing in this study was performed at CHGC. In addition, the restriction sites were analyzed by BioEdit software (http://bioedit.software.informer.com/). The sequence alignments were performed using BLAST (http://blast.st-va.ncbi.nlm.nih.gov/Blast.cgi). Glimmer (http://www.ncbi.nlm.nih.gov/genomes/MICROBES/glimmer_3.cgi) was used to predict open reading frames (ORFs). Transmembrane helices in proteins were predicted by TMHMM Server v.2.0. SecretomeP 2.0 Server was used to predict the secretion of proteins. Conserved domains were determined via Conserved Domain Database (CDD) and Clusters of Orthologous Groups of proteins (COGs) in the NCBI database and InterPro (http://www.ebi.ac.uk/interpro). Orthologous proteins were identified by performing BLAST searches against the NCBI non-redundant protein database and subsequently checked manually.

### Pulsed-Field Gel Electrophoresis (PFGE)

Either 2×10^9^ cells or 20 µL (130 ng/µL) of extracted plasmid DNA were embedded in agarose plugs (1%) (SeaKem Gold agarose; Cambrex, Rockland, ME) and incubated with 0.1 mg/mL proteinase K overnight at 50°C. The plugs were then washed twice with pre-warmed TE buffer, and then digested with either restriction enzyme NotI or BamHI at 37°C or 30°C, respectively, following manufacturer's instructions (New England Biolabs, Ipswich, MA, USA). DNA fragments were separated using a 1% pulse-field agarose gel, which was prepared and ran in 0.5%TBE buffer on a contour-clamped homogenous electric field machine (CHEF-DR III; Bio-Rad, Richmond, CA). The lysates were electrophoresed at 5 V at room temperature for either 5–65 sec for 24 h or for 5–25 sec for 24 h based on the fragment size. Gels were stained in 20 µg/mL ethidium bromide (Sigma, St Louis, MO, USA) for 15 min and destained with distilled water for 30 min. *Salmonella Braenderup* (H9812) from the Institute for Infectious Disease Control and Prevention (Beijing, China) was digested with XbaI (New England Biolabs) and used as the molecular weight size standard.

### Southern hybridization

Total genomic and plasmid DNA were digested with either NotI or BamHI and electrophoresed in a 1% agarose gel. Southern blot hybridization was performed using a DIG luminescent Detection Kit (Roche Diagnostics, Indianapolis, IN, USA) according to the manufacturer's instructions with primers listed in Table 1.

**Table 1:** Primers used in this study.

Probe	Primer ID	Primer sequence (5′-3′)	Product size (bp)	Location
**SpGui1**	**S pGui1-F**	TCGTTGTGCCCAACTCTCAA	486	**pGui1**
	**S pGui1-R**	TCAAATCCGAAAACCCCA		
**SpGui2**	**S pGui2-F**	AAGATCTCCTTTATATAGTGATTTC	496	**pGui2**
	**S pGui1-R**	GTATTTTTATCAATCACAAATAATG		
***dnaA***	**dnaA-F**	GCTCCATCTGCCACAATC	92	**large chromosome**
	**dnaA-F**	GGAATCTTATCGCCACAAGTT		
**P1**	**pGui1-F**	CATCGGTATTAGCGTAAGAG	94	**pGui1**
	**pGui1-R**	AGGAATCATCGGAGTTGT		
**P2**	**pGui2-F**	AATCGCACCACCTAATATC	100	**pGui2**
	**pGui2-R**	TCATCGCAATCGTAGAAC		

### Relative copy number determination by quantitative real-time PCR (qPCR) and next-generation sequencing (NGS)

As mentioned above, total genomic DNA of strain Gui44 including both chromosome and plasmid DNA was sequenced with Hiseq 2000 system. Sequenced reads were aligned to the reference genome (including the two chromosomes and two plasmids) of the strain Gui44 by Bowtie2 using the default parameters [22]. When the reads were exclusively mapped to the simple location of the genome, they were counted by using the idxstats module in SAMtools software [23]. The relative copy number was calculated by: aligned reads numbers× average read length/(corresponding chromosome or plasmid size), as well as by qPCR on an ABI 7500 fast real-time PCR system (Applied Biosystems of Thermo Fisher Scientific Inc., Waltham, MA, USA) with a FastStart Universal SYBER Green Master mix (Roche Diagnostics., Indianapolis, USA) according to manufacturer's instructions. PCR primers were designed using Beacon Designer 7 software (http://www.premierbiosoft.com/molecular_beacons/). Eight-fold serial dilutions of the plasmids from Peasy-T1 (Beijing Transgen Biotech Co., LTD, Beijing, China) and template DNA were used to construct standard curves of all plasmids. Reactions were performed in duplicate in a total reaction volume of 20 µL. A 92 bp fragment of a replication initiation factor (*dnaA*), a single-copy gene in the larger chromosome, was used as a reference gene. A 94 bp fragment designated P1 that is unique to pGui1 and a 100 bp fragment designated P2 which is specific to pGui2 were amplified (all primers used for qPCR are listed in Table 1). The reactions were performed under the following conditions: 10 min at 95°C, followed by 40 cycles of 30 sec at 95°C, 30 sec at 56°C, and 30 sec at 72°C. The experiment was carried out in triplicate and averages were reported. The relative copy number of plasmids was calculated using N_relatives_ = (1+E)^−ΔC^_T_, where E stands for the PCR amplification efficiency and ΔC_T_ was for the difference in threshold cycle number between the reference gene and target gene.

### Nucleotide sequence accession number

The complete nucleotide sequences of pGui1 and pGui2 plasmids have been deposited in GenBank under the following accession number: KF648557 and KF648558.

## Results and Discussion

### Analysis of pGui1 and pGui2 isolated from *L. interrogans* strain Gui44

**Figure 1:**
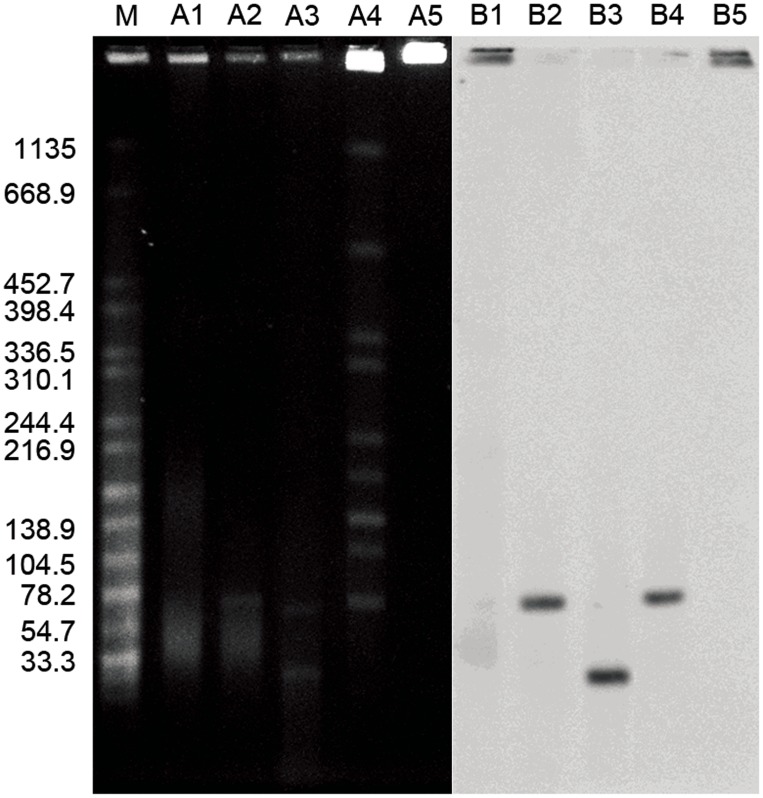
Characterization of pGui1 and pGui2 in the pathogenic *L.interrogans* strain Gui44 by PFGE and Southern blot. PFGE and Southern Blot (using radiolabeled spGui1 probe) of undigested plasmid DNA (lanes A1 and B1); plasmid DNA digested with NotI (A2 and B2); plasmid DNA digested with BamHI (A3 and B3); total genomic DNA digested with NotI (A4 and B4); and undigested total genomic DNA (A5 and B5). The fragment in A2 and B2 indicates the linearized pGui1 (∼78 kb). The corresponding fragment presents in A4 and B4. The larger fragment in A3 indicates the linearized pGui2. The smaller fragment in A4 is one fragment of pGui1 digested with BamHI. *Salmonella Braenderup* (H9812) digested with XbaI and is used as the molecular weight size standard (M).

**Figure 2:**
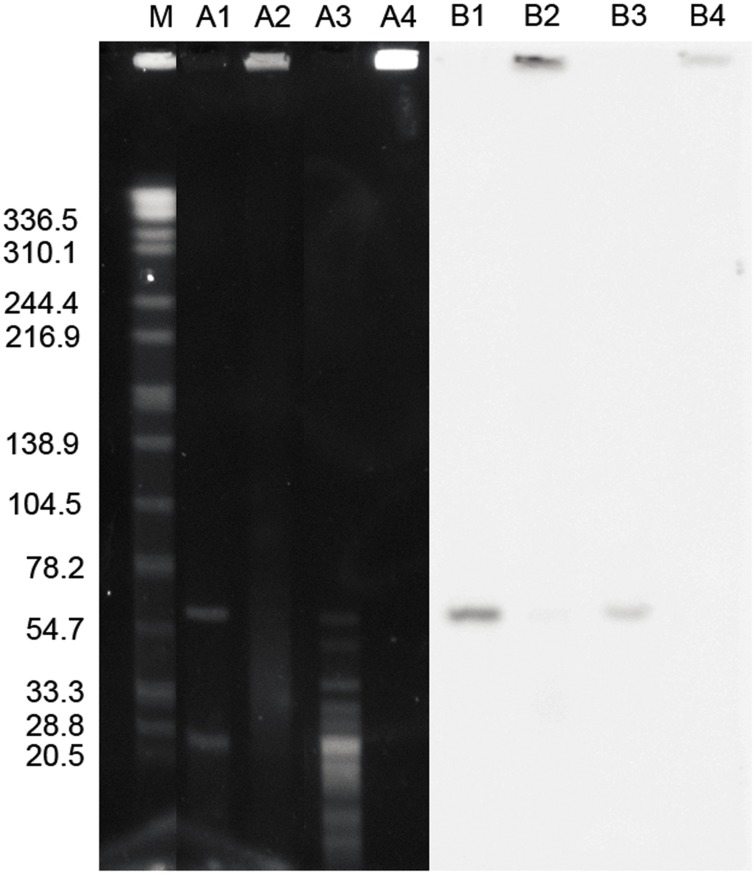
Characterization of pGui2 in the pathogenic *L.interrogans* strain Gui44 by PFGE and Southern blot. PFGE and Southern Blot (using radiolabeled spGui2 probe) of plasmid DNA digested with BamHI (lanes A1 and B1); undigested plasmid DNA (A2 and B2); total genomic DNA digested with BamHI (A3 and B3); and undigested total genomic DNA (A4 and B4). *Salmonella Braenderup* (H9812) digested with XbaI was used as the molecular weight size standard (M). The larger fragment in A1 indicates the linearized plasmid pGui2. The corresponding fragment presents in A3. The smaller fragment in A1 is a fragment of the plasmid pGui1.

*In silico* analysis revealed that the pGui1 has one NotI restriction site and nine BamHI restriction sites, whereas pGui2 only contains a BamHI restriction site but no NotI restriction site. Therefore, following plasmid digestion with BamHI, pGui1 has eight specific fragments of 217, 1903, 2328, 3134, 3947, 19971, 21277, and 22104 bp. Digestion of genomic and plasmid DNA with NotI both identified the presence of pGui1 by a fragment of the expected 78 kb size (Fig. 1). Southern hybridization results using a specific probe SpGui1 suggested that pGui1 was circular and independent of the chromosome (Fig. 1). Digestion of genomic and plasmid DNA using BamHI and Southern hybridization with SpGui2 probe revealed a ∼65 kb fragment (Fig. 2). BamHI digested plasmids hybridized with probe SpGui1 revealed a ∼23 kb fragment (Fig. 1). Collectively, these results confirmed the presence of two circular plasmids in the *L.interrogans* serovar Canicola strain Gui44 with sizes of approximately 78 kb and 65 kb.

### General characteristics of pGui1 and pGui2

pGui1 and pGui2, were found to be 74,981 and 66,851 bp in length with overall GC content of 34.63% and 33.33%, respectively. A summary of the general characteristics is presented in Table 2, and detailed genomic maps are provided in Supplementary Fig.S1. The G+C content of the two plasmids was lower in comparison with the chromosomes of *L.interrogans* strain Gui44 (35.05%, unpublished data), as well as with the third circular replicon p74 (37.47%) previously found in the *L.biflexa* and the extrachromosomal circular plasmid Laicp in strain 56601 (34.64%) [17]. The pGui1 contains 62 protein-coding genes representing a coding percentage of 78.3%, with the majority of the identified genes (56%) encoding hypothetical proteins. In addition, mobile elements (15%), genes related to heavy-metal and drug resistance (10%), and regulatory genes (6%) were also identified. A total of 63 protein-coding genes, comprising 72% of the plasmid, were identified in pGui2 (Table 2). The percentage of genes encoding hypothetical proteins (82%) on pGui2 was higher than on pGui1 (56%). In addition, mobile elements (6%) and regulatory genes (2%) were also identified. Identified genes and their putative functions are listed in Supplementary Table S2 and S3. The high percentage of mobile elements suggests the presence of gene rearrangement. The differences between pGui1 and pGui2 suggest they have different roles in parasite cell function.

**Table 2:** Genome features of pGui1 and pGui2.

Genomic Features	pGui1	pGui2
Size (bp)	74981	66851
G+C content (%)	34.63	33.33
Protein coding (%)	78.32	72.497
Protein-coding genes	62	63
Hypothetical genes	35	52
Gene density (bp per gene)	947	769
Transposases	9	4

### Plasmid stability

As extrachromosomal DNA, plasmids must meet the following criteria if they are to be stably maintained over time in the host cells: (i) execute functions that ensure accurate segregation, (ii) contain effective systems that result in inherited stability, and (iii) be capable of replicating autonomously within a suitable host. Accurate segregation and inherited stability are especially important for the low copy number plasmids [24]. Sequence analysis of pGui1 and pGui2 shows that both plasmids meet these criteria.

In the present study, since we sequenced the genome of strain Gui44 by 454 sequencing and firstly detected the existence of the two novel extrachromosomal elements in 2009, the Gui44 strain has been cultured in EMJH and maintained by serial passages at least once a month in hamsters for the preservation of virulence for more than two years, in 2011, whole genome re-sequencing of plasmid DNA and total genomic DNA of the strain Gui44 were independently performed using Hiseq 2000 system. Although we do not have a precise number of total passages, the two independent sequencing results of plasmid DNA and genomic DNA both confirmed that the two plasmids, pGui1 and pGui2, are stably maintained in the strain Gui44, representing their potential application as novel shuttle vectors in pathogenic *Leptospira*.

Furthermore, the PFGE profiles in the present study following NotI digestion are fully consistent with a previous report in 2012 [25]. Particularly, the same 75 kb fragment of pGui1 was observed, further confirming the stable presence of pGui1. However, due to the absence of NotI restriction site, pGui2 did not appear as a linearized fragment when digested with NotI performed by Tang GP, et al and our study [25].

### The partition system

Low-copy-number plasmids require a segregation mechanism to ensure that equivalent plasmid copies are inherited by each daughter cell during cell division. Several specific mechanisms may be involved in DNA segregation [26], [27]. One such mechanism involves *par* loci, which ensures receipt by every daughter cell, thereby enhancing the inheritance and stability of the plasmid DNA [27], [28]. Genes that may be involved in plasmid segregation were also identified in pGui1 and pGui2. pGui10067, identified in pGui1, encodes a protein that shares high similarity with LA1838, the chromosome partitioning protein *parB* locating on the newly confirmed plasmid Laicp of *L.interrogans* serovar Lai *str.*56601 in our recent study (98/227 aa, 43% identity) [17] and LEP1GSC075_0005, *ParB*-like protein of *L.interrogans str.*Kito (261/261 aa, 100%) in the PATRIC database [29]. Another gene found in pGui1, pGui10068, encodes a protein that exhibits similarities with LA1837, one *parA* family protein encoded by the extrachromosomal plasmid of *str.*56601 (116/258 aa, 45%) [17] and LEPBI_p0001, putative chromosome partitioning protein *ParA* in the plasmid p74 of *L.biflexa* serovar Patoc strain Patoc 1 (Paris) (117/257 aa, 46%) [29]. Similarly, pGui20067 identified in pGui2, shares high similarity with LEP1GSC069_1546, *ParA* nucleotide binding domain protein of *L.interrogans* serovar Canicola *str.* Fiocruz LV133 (229/247 aa, 92%), and its downstream pGui20066, exhibits similarity with LEP1GSC051_0353, *ParB*-like protein in *Leptospira sp.*P2653 (114/235 aa, 49%) [29]. Therefore, as *ParB* is known to be less conserved [14], pGui20066 may constitute the *parB* within the Gui44 strain. These partition systems in pGui1 and pGui2 may play an important role in the plasmids maintenance in the *L.interrogans* serovar Canicola strain Gui44.

### The postsegregational killing system (PSK)

One way organisms maintain a plasmid in a population is through the addiction system also termed plasmid PSK [24]. Although there are several types of plasmid addiction systems, the one found in *L.interrogans* serovar Canicola strain Gui44 consists of toxin and antitoxin genes, most likely encoding a labile antitoxin and a stable toxin. In the toxin/antitoxin system (TA), the daughter cells that contain the addiction system, are able to survive due to the presence of the antitoxin whereas, the daughter cells that lack the plasmid either die or exhibit reduced growth-rate due to the presence of the toxin being produced by the parent cell [24], [26]. The addiction system in cells plays an essential role in the execution of postsegregational killing. In pGui1, pGui10006 and pGui10007 are identical to ppGpp-regulated growth inhibitor MazF of *L.interrogans* serovar Lai *str.*Lai (99/99 aa, 100%) and the transcriptional regulator/antitoxin MazE of *L.interrogans* serovar Lai *str.*Lai (99/99 aa, 100%), respectively [29]. An additional Toxin-Antitoxin system was identified in pGui1, encoded by pGui100011 and pGui10012, which show high similarity with MazF and MazE in *L.borgpetersenii* serovar Hardjo-bovis L550 (103/107, 96% and 77/82, 94%, respectively). MazF is a sequence-specific mRNA endoribonuclease that initiates a programmed cell death pathway, whereas MazE acts as its antagonist [30]. In pGui2, no obvious addiction system was found. However, hypothetical protein pGui20047, displays some similarity with the antitoxin component of repressor protein CI in *Burkholderia multivorans* CGD2M (22/62 aa, 35%) and antitoxin component in *Algoriphagus spp.* PR1 (36/121 aa, 30%). These homologs involved in the plasmid addiction system may contribute to the stable inheritance of pGui1 and pGui2 in the strain Gui44.

### The replication region

Proper plasmid replication depends upon the replication region and replication origin [31]. Replication origins of pGui1 and pGui2 were predicted by GC skew [32] (Supplementary Fig.S1). The most important replication protein (Rep) of low-copy-number plasmid is often found in the vicinity of *ParAB* system [8]. Analysis of pGui1 and pGui2 showed that pGui10066 and pGui20065, both located downstream of *ParAB-*system, share high similarity with LA1839 in the plasmid Laicp as well as in the previously identified genomic island LaiGI which has the ability for autonomous replication in *L.interrogans* serovar Lai strain Lai and serovar Canicola strain HondUtrech as well as *L.biflexa* [8]. The C-terminal regions of pGui10066 and pGui20065 (272–478 aa) share 86% and 88% similarity with the LA1839 protein, respectively. Analysis of the conserved domains in the N-terminal region of the three replication proteins (1–75 aa) revealed a common helix-turn-helix motif, a sequence-specific DNA binding domain characteristic of Rep proteins [31]. Hence, these findings suggest that pGui10066 and pGui20065 are potential replication proteins for pGui1 and pGui2.

### Genes with putative role in virulence

Genes involved in pathogenicity were also identified in both pGui1 and pGui2. pGui10044 encodes one protein that contains six tandem repeat bacterial immunoglobulin-like (Big) domains, which are classical characteristic domain of the leptospiral Ig-like (Lig) proteins [33]. Lig proteins have been shown to contribute to microbial adhesion of host mammalian cell and initiate infection [34]. Bioinformatic analysis indicates that pGui10044 is a secretory protein with a transmembrane helix, suggesting that it may be involved in *Leptospira*-host interaction. pGui20059 possesses a rearrangement hotspot domain (RHS), which appears to be involved in ligand binding and activates the inflammasome [35], [36]. Based on Interpro analysis, pGui20061 shows features related to VCBS domain (Repeat domain in *Vibrio*, *Colwellia*, *Bradyrhizobium* and *Shewanella*) that was found in some pathogenic bacteria and played a very important role in pathogen adhesion to host cells. The exact function of these proteins deserves further investigations.

### Relative plasmid copy numbers

Whole genome sequencing using total genomic DNA of strain Gui44 generated a total of 26,589,548 reads and nearly 2 Gb high-quality filtered sequences. The numbers of aligned reads and genomic coverage are 23,612,793 and 558.0599 (larger chromosome), 2,014,331 and 564.8649 (small chromosome), 392,726 and 523.7674 (pGui1), 322,523 and 482.4505 (pGui2), respectively. The relative ratio of reads to length of the large chromosome, small chromosome, pGui1 and pGui2 sequenced are 5.5806, 5.6486, 5.2377, and 4.8245, respectively, indicating the closely similar relative copy number.

**Figure 3:**
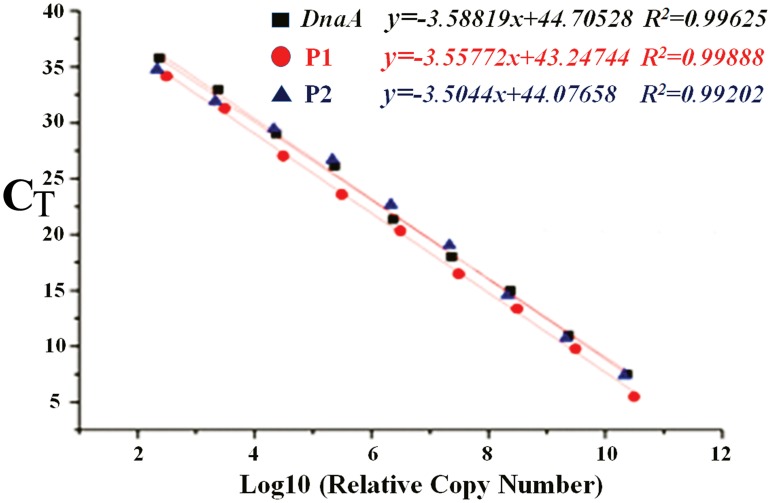
Relative copy number of large chromosome (*DnaA*), pGui1 (P1) and pGui2 (P2) as determined by real-time quantitative PCR. The standard curves obtained from eight-fold DNA dilutions were linear (*R^2^*>0.99) in the range tested.

In order to more precisely determine the relative copy numbers of the two plasmids, the qPCR method was applied. As shown in Figure 3, the standard curves obtained for *DnaA* (larger chromosome), P1 (pGui1) and P2 (pGui2) are linear (*R^2^*>0.99) in the range tested with slopes of 3.58819, 3.55772, and 3.50440, respectively. In addition, the intercepts of *dnaA*, P1 and P2 are 44.70528, 43.24744 and 44.07658, with Ct values of 13.71492, 15.20904 and 14.09529, respectively. Taken together, these results indicate that the relative copy number of pGui1 and pGui2 is about one copy per chromosome. Hence, we speculate that it is difficult to isolate and extract plasmid DNA in pathogenic *Leptospira* because of the low plasmid copy number.

### Comparison of the two plasmids against the extrachromosomal plasmid Laicp of *L. interrogans* str.56601

**Figure 4:**
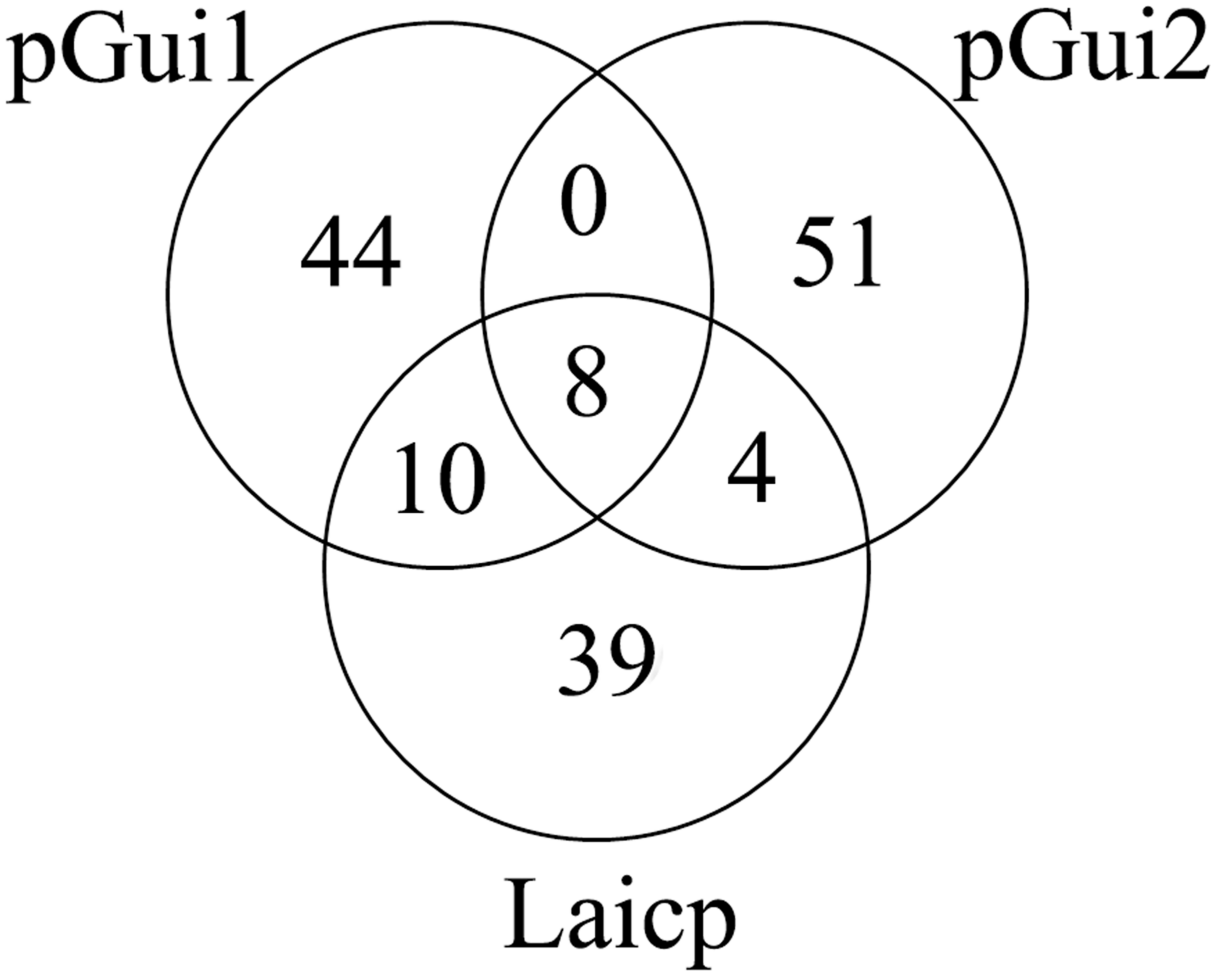
Venn diagram detailing the unique and common gene complements among the three plasmids pGui1, pGui2 and Laicp. Eighteen orthologs in pGui1 and twelve orthologs in pGui2 are common with Laicp. Eight genes are common in the three plasmids. pGui2 possesses the largest number of unique genes (51) among the three plasmids. And 44 genes are unique to pGui1. (Supplementary Tables S1, S2, S3).

Sequence comparisons showed that the Laicp has 18 orthologs in pGui1 (Fig. 4). Six (33%) of these have no known function and six (33%) are transposases. In addition, Toxin-antitoxin proteins (two), transcriptional regulator (one), plasmid partition proteins (two) and replication origin protein (one) were identified on pGui1. Additionally, twelve common orthologs between pGui2 and Laicp were identified. The majority (8/12) of them are hypothetical proteins, whereas two are transposases, as well as one replication origin protein and one AraC family transcriptional regulator. The number of orthologs found in pGui1 is larger than in pGui2. Eight genes were common to all three plasmids (Supplementary Table S1). The transposase, which belong to the transposase 31 superfamily, is distributed widely in *Leptospira* species as confirmed by BLAST searches against the NCBI non-redundant protein database. Orthologs of LA1827 contain the PDDEXK domain, which belongs to the nuclease superfamily, and is identified in several *Leptospira* species. Global sequence comparisons demonstrate that the similarity between pGui1 and Laicp is greater than similarity between pGui2 and Laicp.

### Conclusion

In this report, we present two novel plasmids, pGui1 and pGui2, found in one pathogenic *L.interrogans* strain Gui44. The sequence analysis shows that the two plasmids possess a high percentage of novel genes (56% and 82%, respectively), transposases, integrases and so on which may contribute to the strain's virulence and survival mechanism necessary for persistent infection in different ecological environments and mammalian hosts. Knowledge of the different genomic composition among pathogenic *Leptospira* may deepen our understanding of the discrepancies in their adaptation and pathogenesis. Thus far, at genome level, there was no other report on the successful isolation and characterization of two circular plasmids from pathogenic *Leptospira*. We speculate that this is most likely due to high genomic diversity, low copy number, fastidious growth requirements, slow bacterial growth rate in both solid and liquid media and the lack of suitable plasmid extraction methods. Until recently, the absence of available genetic tools has severely impeded our understanding of pathogenic *Leptospira* species. The demonstrated stability of these two plasmids suggests their potential use for future genetic studies on *Leptospira*, leading to an improved understanding of virulence and gene dissemination in *Leptospira* spp.

## Supporting Information

Figure S1 **Genomic maps of pGui1 and pGui2 plasmids.** Functional information for pGui1 and pGui2 is presented (replicon sizes are shown in bp). The outside circle (circle 1) represents the genomic sequence. The arrows (red) identify the replication protein. OriCs were predicted by GC skew method. Inner circles (circle 2 and circle 3) represent predicted protein coding regions (forward and reverse strands, respectively). Functional COG categories are delineated by default color. GC content is depicted in circle 4, and GC skew is depicted in circle 5. GC content deviations from the genomic average were calculated by using a window of 500 bp in steps of 100 bp.
(TIF)

Table S1 **Common genes among pGui1, pGui2 and Laicp.**
(XLSX)

Table S2 **Unique genes of pGui1 compare to pGui2 and Laicp.**
(XLSX)

Table S3 **Unique genes of pGui2 compare to pGui1 and Laicp.**
(XLSX)
