# Supplementary figures and images for: Isolation and Characterization of Two Novel Plasmids from Pathogenic Leptospira interrogans Serogroup Canicola Serovar Canicola Strain Gui44
Source: PLoS Negl Trop Dis. 2014 Aug 21;8(8):e3103. doi: 10.1371/journal.pntd.0003103 (PMC4140679; doi:10.1371/journal.pntd.0003103)

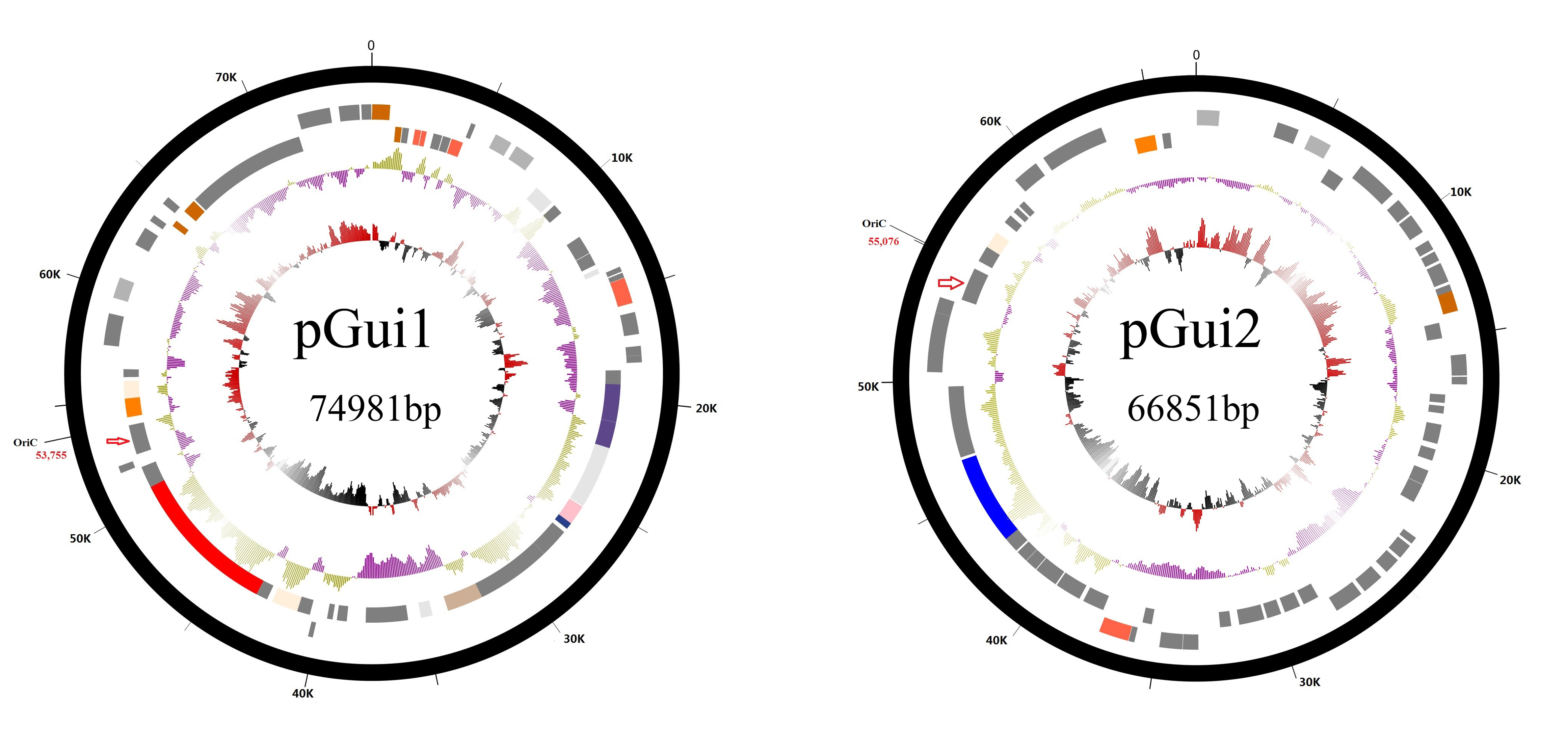

Supplement: Figure S1 — Genomic maps of pGui1 and pGui2 plasmids. Functional information for pGui1 and pGui2 is presented (replicon sizes are shown in bp). The outside circle (circle 1) represents the genomic sequence. The arrows (red) identify the replication protein. OriCs were predicted by GC skew method. Inner circles (circle 2 and circle 3) represent predicted protein coding regions (forward and reverse strands, respectively). Functional COG categories are delineated by default color. GC content is depicted in circle 4, and GC skew is depicted in circle 5. GC content deviations from the genomic average were calculated by using a window of 500 bp in steps of 100 bp. (TIF) [file pntd.0003103.s001.tif]
